# Early Disruption of Extracellular Pleiotrophin Distribution Alters Cerebellar Neuronal Circuit Development and Function

**DOI:** 10.1007/s12035-015-9450-5

**Published:** 2015-09-24

**Authors:** M. M. Hamza, S. A. Rey, P. Hilber, A. Arabo, T. Collin, D. Vaudry, D. Burel

**Affiliations:** 1 INSERM U982, Laboratory of Neuronal and Neuroendocrine Differentiation and Communication, University of Rouen, 76821 Mont-Saint-Aignan cedex, France; 2 Institute for Research and Innovation in Biomedicine (IRIB), Normandie University, Mont-Saint-Aignan, France; 3 CNRS-UMR 8118, Laboratory of Cerebral Physiology, University Paris Descartes, 75006 Paris, France; 4 EA 4700, Laboratory of Psychology and Neuroscience of Cognition, University of Rouen, 76821-cedex Mont-Saint-Aignan, France; 5 UFR sciences et techniques, University of Rouen, 76821-cedex Mont-Saint-Aignan, France

**Keywords:** Pleiotrophin, Cerebellum, Neurodevelopment, Purkinje cells, Locomotor behavior

## Abstract

The cerebellum is a structure of the central nervous system involved in balance, motor coordination, and voluntary movements. The elementary circuit implicated in the control of locomotion involves Purkinje cells, which receive excitatory inputs from parallel and climbing fibers, and are regulated by cerebellar interneurons. In mice as in human, the cerebellar cortex completes its development mainly after birth with the migration, differentiation, and synaptogenesis of granule cells. These cellular events are under the control of numerous extracellular matrix molecules including pleiotrophin (PTN). This cytokine has been shown to regulate the morphogenesis of Purkinje cells ex vivo and in vivo via its receptor PTPζ. Since Purkinje cells are the unique output of the cerebellar cortex, we explored the consequences of their PTN-induced atrophy on the function of the cerebellar neuronal circuit in mice. Behavioral experiments revealed that, despite a normal overall development, PTN-treated mice present a delay in the maturation of their flexion reflex. Moreover, patch clamp recording of Purkinje cells revealed a significant increase in the frequency of spontaneous excitatory postsynaptic currents in PTN-treated mice, associated with a decrease of climbing fiber innervations and an abnormal perisomatic localization of the parallel fiber contacts. At adulthood, PTN-treated mice exhibit coordination impairment on the rotarod test associated with an alteration of the synchronization gait. Altogether these histological, electrophysiological, and behavior data reveal that an early ECM disruption of PTN composition induces short- and long-term defaults in the establishment of proper functional cerebellar circuit.

## Introduction

The cerebellum is an important region of the central nervous system which plays a major role in the control of equilibrium, motor coordination, and posture [1, 2]. Indeed, the cerebellar cortex is able to integrate various sensory inputs to generate appropriate complex voluntary movements [3, 4]. This structure also participates to numerous cognitive processes such as language development, spatial and motor learning [5–7], as well as emotional responses, such as anxiety, fear, and possibly pleasure [8–10].

All the cerebellar functions are directly dependent of a harmonious ontogenesis, leading to appropriate connections of Purkinje cells. In mouse (as in human), the development of the cerebellum mainly occurs during the postnatal period. Thus, at birth (P0), mouse cerebellum consists of three layers. The Purkinje cell layer (PL) includes Purkinje neurons and Bergmann glia, which develop respectively their dendritic tree and radial fibers in the molecular layer (ML). At the surface, the external granular layer (EGL), which corresponds to a secondary germinative zone, contains the granule cell precursors (GCP) [11]. After their final division, granule cells migrate radially through the ML and the PL along the Bergmann fibers to progressively create the internal granular layer (IGL) [12]. In the IGL, granule cells complete differentiation to establish active synapses between their axons (the parallel fibers) and the dendritic tree of Purkinje cells. Two main afferents also contact the cerebellar cortex during the first postnatal weeks. Firstly, the mossy fibers, arising from various brain regions, reach the cerebellum and establish stable synapses with granule cells between P4 and P7. Secondly, climbing fibers which exclusively originate from the inferior olive [13] project to the ML, leading to a mono-innervation of Purkinje cells [14].

From P21, the EGL disappears resulting in a mature cerebellar cortex organized in three layers, named ML, PL, and granule cell layer (GL) at adulthood. Purkinje cells receive direct glutamatergic inputs from the climbing fibers, as well as indirect excitatory messages from mossy fibers via the parallel fibers [15]. Then, these neurons, which represent the only output of the cerebellar cortex, integrate information from multiple brain regions and send an appropriate feedback to deep cerebellar nuclei. This elementary circuit is finally modulated by interneurons. In the ML, stellate and basket cells directly inhibit the Purkinje cells, while, in the IGL, GABAergic Lugaro cells and excitatory unipolar brush cells innervate granule cells to adjust the mossy fiber signal.

The development of the cerebellar cortex is under the control of a complex cellular microenvironment, consisting of growth factors, neuropeptides, neurotransmitters, and components of the extracellular matrix (ECM). Among them, pleiotrophin (PTN) has been recently shown to regulate the cerebellar histogenesis in mouse [16]. This cytokine is an 18-kDa protein of the ECM highly expressed during the postnatal cerebellar development [16, 17]. Its 136-amino acid sequence is conserved throughout various species and contains numerous lysine residues responsible for its high stability [17, 18]. In rodents, PTN participates, ex vivo and in vivo, to the morphogenesis of Purkinje cells via a chondroitin sulfate proteoglycan named protein tyrosine phosphatase ζ (PTP-ζ) [16, 19, 20]. Moreover PTN controls the rate of radial migration of GCP through the ML as well as their apoptosis within the IGL [16].

All these results strongly suggest that PTN could play a major role in the setting of cerebellar circuits during the postnatal development. Thus, we decided to investigate the influence of PTN on the neuronal activity of Purkinje cells and locomotor functions during the postnatal development of the mouse cerebellum.

## Materials and Methods

### Animals

Animals used in this study were wild-type mice of both sexes with B6/CBA background aged from P4 to P90. All animals were from a colony raised at the University of Rouen in an accredited animal facility (approval D.76-451-04), in compliance with the European directive 2010-63 and according to the French recommendations for the care and use of laboratory animals. Young mice were kept in group cages with woodchip bedding under a 12-h light/dark cycle (light on at 07:30 a.m.) with free access to food and tap water. When weaning, animals were grouped by sex and litter. Experiments were conducted under the supervision of authorized investigators (D.B., D.V.) in accordance with the ethical committee (CENOMEXA No. 54: approval number N/01-12-11/24/12-14).

### Materials

The normal donkey serum (NDS), the 4′,6′-diamidino-2-phenylindole (DAPI), and the anti-calbindin antibody produced in mouse (C9848) were purchased from Sigma-Aldrich (Saint Quentin Fallavier, France). The anti-glutamate receptor delta 2 (GluRδ2) antibody developed in goat was obtained from Santa Cruz (Le Perray en Yvelines, France). The anti-calbindin (AB1778), anti-VGlut1 (MAB5502), and anti-VGlut2 (AB2251) antibodies, produced respectively in rabbit, mouse, and guinea pig, as well as Cy3-conjugated donkey anti-guinea pig (DAGp) were from Millipore (Molsheim, France). Alexa 488-, Alexa 568-, or Alexa 546-conjugated donkey anti-goat (DAG), anti-mouse (DAM), or anti-rabbit (DAR) secondary antibodies were purchased from Invitrogen (Cergy Pontoise, France). Recombinant human PTN expressed in *Spodoptera frugiperda* insect cells came from Calbiochem (VWR, Strasbourg, France). Drugs used for electrophysiological recording were from Tocris Bioscience (Bristol, UK) or Ascent Scientific (Weston-Super-Mare, UK).

### Subarachnoidal Injections of Pleiotrophin

Injections were realized at P4 and P6 in the subarachnoid space at the surface of the cerebellum, as previously detailed in Allais et al. [21]. Briefly mice were anesthetized by inhalation of 32 % isoflurane for 30 s as described in Drobac et al. [22]. Then animals were placed on a polystyrene matrix and held with a claw. Injections were carried out with a 5-μl graduated and elongated glass capillary (Harvard Apparatus, Les Ulis, France) fixed to a stereotaxic electrode holder with movement in three directions (David Kopf Instrument, Düsseldorf, Germany) connected to a 10-ml syringe by a silicon catheter (Harvard Apparatus). Injections (3 μl of NaCl 0.9 % or PTN at 10^−8^ M) were performed 5 mm behind the left ear after perforation of cranial cartilage. After injection, the mice were warmed up under a lamp and then put back in their cage.

### Immunohistochemistry

Mice were anesthetized by peritoneal injection of pentobarbital (80 mg/kg) before intracardiac perfusion firstly with NaCl 0.9 % to rinse blood vessels and then with paraformaldehyde (PFA) 4 % diluted in NaCl 0.9 % to fix tissues. Brains were rapidly dissected, postfixed for 3 h in PFA 4 %, and sectioned into frontal 40-μm-thick slices with a vibrating blade microtome VT1000S (Leica Microsystem, Nanterre, France) before immunolabeling. Tissues were permeabilized and saturated with 1 % BSA and NDS diluted at 1:50 in phosphate-buffered saline (PBS) containing 0.3 % Triton X-100 for 90 min at room temperature and then incubated with primary antibodies against GluRδ2, VGlut1, VGlut2, and/or calbindin diluted (1:500) in the same buffer for 3 days at 4 °C. After three 10-min washes in PBS, the sections were incubated at room temperature for 90 min with the appropriate secondary antibody diluted at 1:200 in PBS containing 0.3 % Triton X-100. The tissues were rinsed three times for 10 min in PBS and then labeled for 1 min with DAPI (2 μg/ml) for nucleus counterstaining. Finally, the slices were rinsed twice with PBS and mounted with Mowiol to perform image acquisition with a SP2 acoustico-optic beam splitter confocal laser scanning microscope (Leica Microsystems). The analysis of the co-localization between calbindin and GluRδ2, VGlut1, or VGlut2 and the construction of the three-dimensional images were realized by using Imaris software (Bitplane, Zurich, Switzerland). All three-dimensional reconstructions were done with the same threshold settings, and each image corresponds to a 10-μm section with 1-μm step z-stacks (1024 × 1024 focal planes) acquired by using a ×40 objective.

### Electrophysiological Experiments

Vermis parasagittal (200 μm) or transverse (300 μm) slices were cut from 9–12-postnatal-day-old (P9–12) mice using a vibrating blade microtome (Leica VT 1200S, Leica Microsystems) in ice-cold bicarbonate buffered saline (BBS) solution containing (in mM) the following: 152 NaCl, 2.5 KCl, 2 CaCl_2_, 1 MgCl_2_, 1.25 NaH_2_PO_4_, 26.2 NaHCO_3_, and 10 glucose (saturated with 95 % O_2_–5 % CO_2_), pH 7.3. Slices were incubated at 34 °C for 45 min in BBS solution and stored at room temperature for further use. For recording, slices were installed in a chamber (1.2 ml approx. vol.) and perfused with BBS solution (2.5 ml/min) at room temperature. Purkinje cells were identified using an Axio Examiner A1 upright microscope (Zeiss, Oberkochen, Germany) with Normarski differential interference contrast optics through a water immersion ×60 objective (0.90 numerical aperture), and they were maintained at −64 mV in the whole-cell configuration of the patch clamp using an EPC-9 amplifier (HEKA Electronik, Darmstadt, Germany). The gigaseals were obtained using borosilicate pipettes of 2–3 MΩ when filled with the following intracellular solution (in mM): 150 KCl, 4.6 MgCl_2_, 0.1 CaCl_2_, 10 Hepes-K, 1 EGTA-K, 0.4 Na-GTP, and 4 Na-ATP, pH 7.3. Series resistance was usually comprised between 3 and 8 MΩ and compensated up to 80 %. To investigate Purkinje cell passive properties, a hyperpolarizing voltage pulse of 20 ms from the holding potential (−64 mV) to −84 mV was performed. Current traces were filtered at 6.67 KHz and digitized at a sampling rate of 50 μs per point. During synaptic activity recording, either NBQX (5 μM; Ascent Scientific, Weton-super-Mare, UK), to block excitatory postsynaptic transmission (EPSC) mediated by AMPA receptors, or gabazine (10 μM; Tocris Bioscience Bristol, UK) to eliminate inhibitory postsynaptic transmission (IPSC) through GABA_A_ receptors were added to the bath. Synaptic currents were filtered at 1.3 kHz and sampled at a rate of 250 μs per point. Capacitive currents in Purkinje cells were fitted by a double exponential as previously reported [23] to infer their fast and slow components’ time constants and amplitude coefficients. Analyses were performed off-line with routines running under the Igor Pro programming environment (WaveMetrics Inc., Lake Oswego, USA) including NeuroMatic (developed by Jason Rothman, http://www.neuromatic.thinkrandom.com/) for detection and analysis of EPSCs and IPSCs.

### Developmental Behavior Investigations

Behavioral assessments were performed in blind condition between 10:00 a.m. and 5:00 p.m. on 21 animals (*n* = 10 for control, *n* = 11 for PTN). Thirty minutes before the beginning of each test, animals were familiarized to the testing room. The apparatus were cleaned with an alcohol solution (10 % ethanol) and dried before testing each animal. As an index of correct general development, the weight of each mouse was measured between 09:00 a.m. and 10:00 a.m., daily from P0 to P21 and then every week until the end of the experiment session. Tests are presented below in their chronological order.

#### Turn Over Test

In this test, pups were maintained in a supine position for 1 s and then released. The performance corresponded to the time spent to recover a complete prone position (four paws in contact with the plane testing surface with a cutoff period fixed at 60 s). It was recorded daily during two consecutive trials spaced by a 30-s interval between P8 and P15.

#### Hanging Test

Pups were hanging by their forepaws on a 25-cm horizontal stretched string (diameter about 0.3 mm) placed 10 cm above the hand of the experimenter (to caution the fall). The latency before falling was measured (maximum time 60 s). The animals were subjected to two trials spaced by a 3-min interval every day from P8 to P15.

#### Straightening Test

The straightening test was used to investigate motor coordination and muscular strength of P21 mice. Each mouse was placed in a vertical transparent cylinder (15-cm diameter, 50-cm high) opened on the top. We measured the number of rears (when the animal stands on its hind legs with its two forepaws in contact with the wall) for 3 min.

### First Set of Adulthood Behavioral Investigations

The first set of adult behavioral tests has been realized at P90 with the 21 animals used for developmental investigations (*n* = 11 for control, *n* = 10 for PTN; Table 1).

**Table 1:** First set of adult behavioral investigations

	D1	D2	D3	D4	D5
2:00 p.m.	Muscular strength	Stationary beam	Rotarod test		Unstable platform test

*Dx* day of training

#### Stationary Beam

The stationary beam apparatus was used to investigate the equilibrium abilities and the motor coordination in mice [24]. Each mouse was placed on a wooden beam (1-m length, 2.5-cm width, and 2.5-cm thickness) situated at a height of 45 cm above a thick foam pad. The latency before falling and the walking time were measured in a single trial with a 120-s cutoff period.

#### Rotarod Test

The mice were submitted to the rotarod test to evaluate their motor coordination [25]. The apparatus (BioSeb®, Vitrolles, France) consisted of a 3-cm diameter horizontal rotating cylinder suspended 25 cm above the landing platform. The cylinder was divided by white acrylic disks into five sections of 5 cm each, allowing simultaneous tests of mice. The mice were placed on the top of the revolving beam at a fixed speed of 17 rotations per minute (rpm). Latencies before falling were measured in three-trial sessions with a 30-min intertrial interval and a 120-s cutoff period. When the mice rotated passively for two complete turns, their trial was counted as a fall.

#### Unstable Platform Test

We investigated the equilibrium capabilities on static conditions by using the unstable platform test [26]. It consisted of a circular platform (8.5-cm diameter, 16-g weight) screwed to a 45-cm-high vertical axis which could tilt by 30° in any direction. A nonskid covering was fixed on this platform to avoid sliding and grasping. The mice were individually placed at the center of the platform which was in a horizontal position. From this moment, their movements could provoke tilting of the board. To avoid falling, mice must distribute muscular strength in their limbs and body in an adapted fashion. Each animal was subjected to four trials with a 5-min intertrial interval. Latency before falling was recorded with a maximum time fixed to 120 s. We also considered that animals fall when their two hind paws pass outside the platform or clung to the edge by their forepaws.

### Second Set of Adulthood Behavioral Investigations

In the second set of behavioral tests, 17 animals were used (*n* = 7 for control, *n* = 10 for PTN). As they were not subjected to perinatal manipulation, this novel set of tests aims at further clarifying the effect of PTN administration at adulthood on motor coordination and emotional reactivity without postnatal handling (Table 2).

**Table 2:** Second set of adult behavioral investigations

	D1	D2	D3–D4	D5	D6–D7	D8–D11
10:00 a.m.	Actimetry test	Parallel rod floor test		CatWalk test		Rotarod test
2:00 p.m.	Muscular strength test	0-maze test				

*Dx* day of training

#### Actimetry Test

Spontaneous locomotor activity was assessed by using a Versamax animal activity monitor (AccuScan Instruments, Columbus, OH, USA). Between 10:00 a.m. and 11:00 a.m., animals were placed randomly and individually into Plexiglas actimeter chambers (40 × 40 × 30 cm) and their activity was monitored in the darkness for 60 min. Evolution of distance traveled and walking time were recorded during this period.

#### Parallel Rod Floor Test

The animals were submitted to parallel rod floor test for 15 min to evaluate their locomotor activity and coordination. The apparatus (Stoelting Europe, Dublin, Ireland) was enclosed in a clear Plexiglas chamber (20 × 20 × 28 cm) where 1.6-mm diameter rods are spaced 6 mm apart and elevated 1 cm above the floor made of a metal plate. In this assay, each mouse was required to walk freely for 15 min. Foot slips were detected when a paw contacted a metal plate and scored by the software Any-Maze 4.83w (Stoelting). Furthermore, the total distance and the walking time were also measured by the ANY-maze tracking system.

#### 0-maze Test

The 0-maze test was used to evaluate the anxiety-related behaviors. This apparatus (Med Associate Inc., St. Albans, VT, USA) was a 46-cm diameter circle located 50 cm above the floor and consisted in alternating two open and closed 6-cm width runways. Mice were first placed in one of the closed runways and were allowed to freely explore maze for 5 min in a single session. The animals were tracked by EthoVision XT 8.5 software (Noldus Information Technology, Wageningen, Netherlands). The time spent in the open and closed arenas (when the center point, the nose, and the tail were simultaneously in the same area) was then measured. We also recorded the time spent between the open and closed part of the devise.

#### CatWalk Test

The gait of mice was analyzed by using the CatWalk XT 9.1 system (Noldus Information Technology, Wageningen, Netherlands). This apparatus consisted of an enclosed walkway (130 × 6.5 × 15 cm) with a glass plate and a speed video recording camera. The mice must cross the walkway from the start to their cage. A correct run was defined as one complete crossing of the walkway without interruption. Three correct runs were analyzed for each mouse. After identification of individual footprints, an automated analysis of comparative paw statistics was realized to determine the stride length, the duty cycle, and the step cycle.

#### Constant Accelerating Rotarod Test

The apparatus Rotamex 5 (Columbus Instruments, Columbus, OH, USA) consisted of a 3-cm diameter horizontal rotating cylinder suspended 46 cm above a bedding of sawdust. The cylinder was divided by white acrylic disks into four sections of 9.3 cm each, allowing simultaneous tests of mice. The speed of rotation was increased at a constant rate of acceleration (0.4 rpm/s) from 0 to 17 rpm the first day. Then the maximum speed to reach was elevated to 2 rpm each day (with the same acceleration). Maximum speed was fixed at 23 rpm in the fourth training day. The reached speed before falling was measured in three trial sessions per day with a 30-min intertrial interval.

### Statistical Analysis

Data from turn over test, hanging, exploratory activity, and accelerated rotarod test were assessed using a two-way analysis of variance (ANOVA) with treatment and repeated measures as principal factors, followed by post hoc comparison. Because of unequal variance, the other results were analyzed by the unpaired *t* test (electrophysiological results) or Mann-Whitney test (histological and behavioral results). For all analysis, *p* < 0.05 was considered as significant.

## Results

### Effect of PTN on Purkinje Cell Morphology

**Fig. 1:**
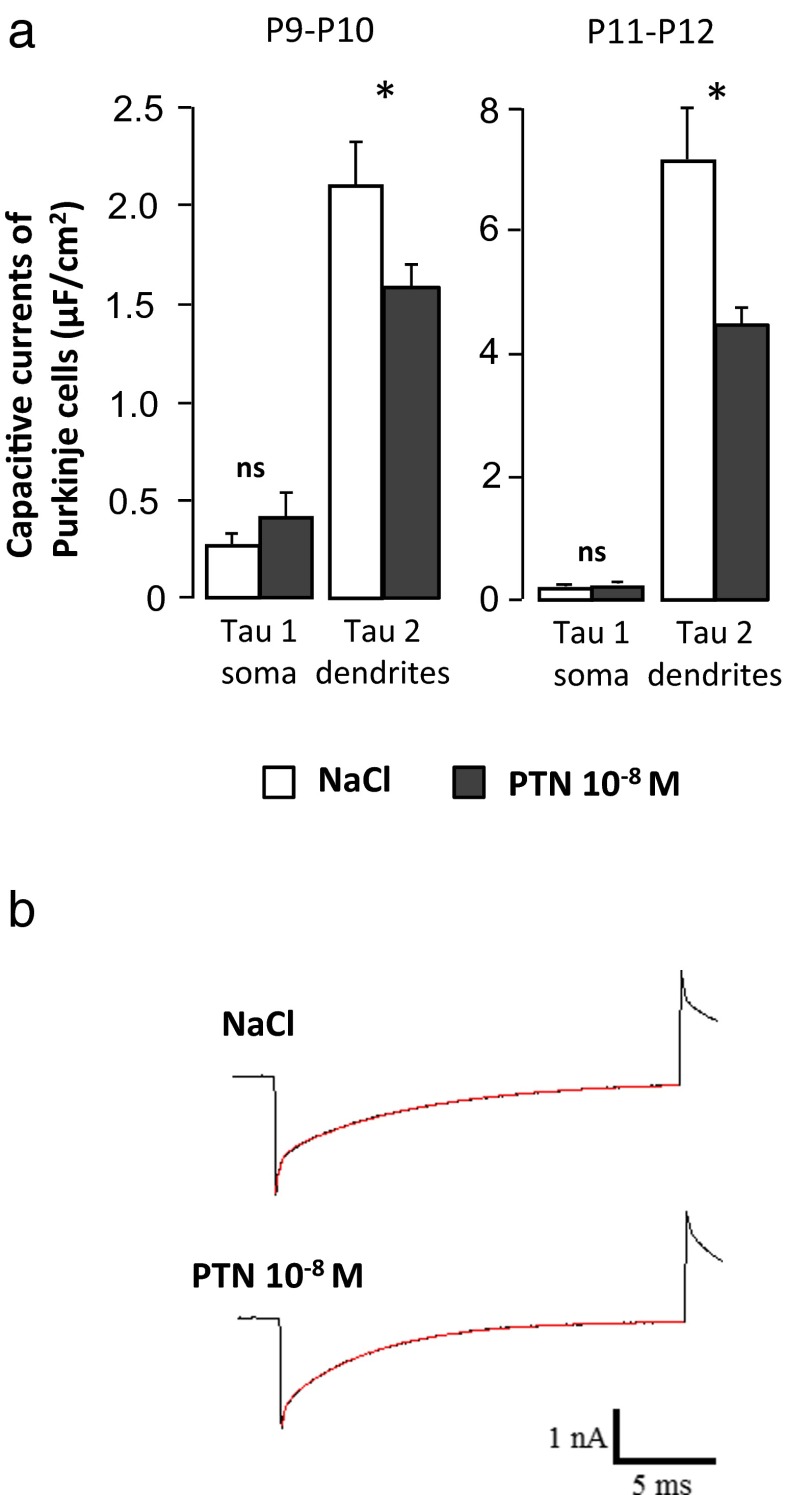
Effect of subarachnoidal injections of PTN on the capacitive currents in Purkinje cells during development. **a** Measurement of Purkinje cell capacitive currents in the fast component, representing the soma (Tau 1), and in the slow component, corresponding to the dendrites (Tau 2) in control (NaCl, *white bars*) and PTN-treated (PTN 10^−8^ M, *gray bars*) mice. Each value represents the mean ± SEM of the measurement of at least 10 cells. **b** Illustration of current responses to 20-mV hyperpolarization voltage pulses from the holding potential of −64 mV. A double exponential fit of the current is observed in red in control (NaCl) and PTN-treated (PTN 10^−8^ M) mice. *Px* postnatal day *x*. *p < 0.05; *ns* not significant

To confirm the early effect of PTN on the dendritic tree of Purkinje cells [16], capacitive currents were recorded in whole cell under voltage clamp condition from sagittal cerebellar slices at the level of the simplex lobule. They were generated by an hyperpolarized step of 20 ms to −84 mV from the holding potential, and current responses were fitted by a double exponential. In this current model described in details by Llano et al. [23], fast and slow components of capacitive current responses were interpreted with an equivalent electrical circuit in two compartments corresponding firstly to the soma and proximal dendrites (perikarya component) and secondly to the rest of the dendritic tree (dendritic component). In P9–P10 control mice, Purkinje cell capacitive current responses displayed a fast component with a time constant (tau 1) of 0.29 ± 0.05 ms and a slow component with a time constant (tau 2) of 2.16 ± 0.19 ms (*n* = 21 cells from five animals; Fig. 1a, b). No significant difference was observed for tau 1 between control and PTN-treated mice (for PTN mice, 0.44 ± 0.08 ms; *n* = 10 cells from five animals). Interestingly, tau 2 was significantly smaller in PTN-treated mice in comparison with control animals (1.63 ± 0.13 ms; *p* < 0.05; *n* = 10 cells from five animals). The same results were observed with Purkinje cells from P11–P12 animals (for control mice, *n* = 9 cells from one animal; for PTN mice, *n* = 15 cells from two animals; Fig. 1a, b).

In adult mice, the immunohistochemical analysis of calbindin labeling indicates that there is no difference in the morphology of the dendritic trees of Purkinje cells in PTN-treated mice (*n* = 3, data not shown).

### Effect of PTN on the Purkinje Cell Afferents

**Fig. 2:**
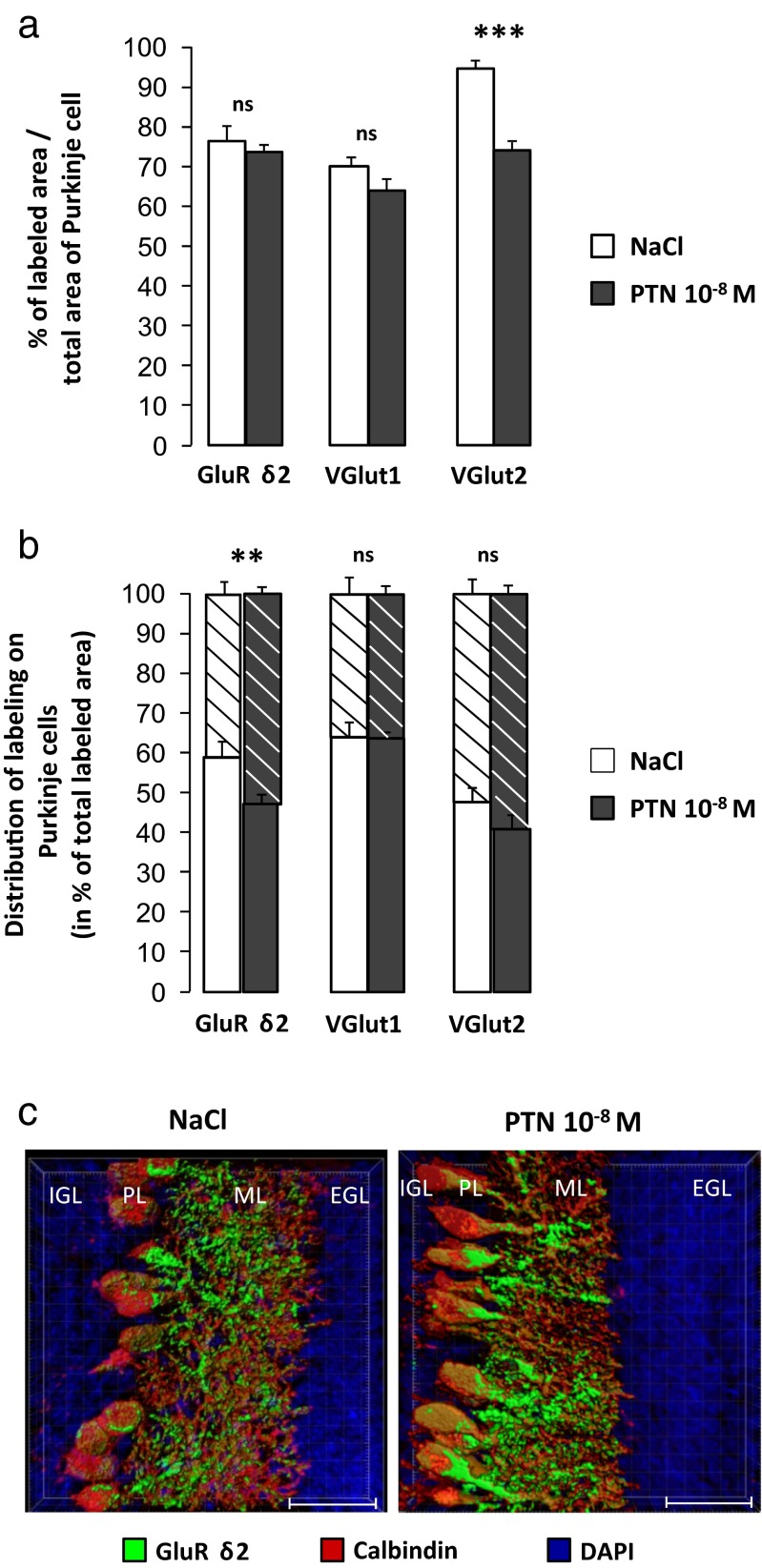
Effect of subarachnoidal injections of PTN on Purkinje cell afferents during development. **a** Analysis of the GluRδ2-, VGlut1-, and VGlut2-labeled areas in percentage of co-localization with calbindin labeling on Purkinje cells in control (NaCl, *white bars*) and PTN-treated (PTN 10^−8^ M, *gray bars*) mice at postnatal day 10. Each value represents the mean ± SEM of the analysis of at least 450 Purkinje cells from three (NaCl) or four (PTN 10^−8^ M) different animals. **b** Distribution, in percentage of total labeling, of the GluRδ2, VGlut1, and VGlut2 labeling between the soma compartment (*striped bars*) or in the dendrite component (*solid bars*) in control (NaCl, *white bars*) and PTN-treated (PTN 10^−8^ M, *gray bars*) mice at postnatal day 10. Each value represents the mean ± SEM of the analysis of at least 450 Purkinje cells from three (NaCl) or four (PTN 10^−8^ M) different animals. **c** Three-dimensional representation of the GluRδ2 distribution (in *green*) on calbindin-labeled Purkinje cells (in *red*) in control (NaCl, *left*) and PTN-treated (PTN 10^−8^ M, *right*) mice at postnatal day 10. Nuclei are counterstained with DAPI in blue. Scale bar = 30 μm. *EGL* external granular layer, *IGL* internal granular layer, *ML* molecular layer, *PL* Purkinje cell layer, P*x*, postnatal day *x*. **p < 0.01; ***p < 0.001; *ns* not significant

To determine if the PTN-induced alteration of Purkinje cells causes a default of afferents, the distribution of GluRδ2, VGlut1, and VGlut2 has been studied at the level of the simplex lobule. The data were obtained on three or four control and PTN-treated P10 mice, respectively, by analysis of at least 450 Purkinje cells from four different slices. Our results show that the rate of co-localization between GluRδ2 and calbindin labeling is similar in both groups of animals, but the distribution of the GluRδ2 is significantly different in the PTN-treated mice with an increase of the immunoreactivity in the perikarya component of Purkinje cells (*p* < 0.01; Fig. 2a–c). In parallel, the VGlut2 labeling is decreased in PTN-treated animals and this diminution is equally distributed between the soma and the dendritic tree of Purkinje cells (*p* < 0.001; Fig. 2a, b). PTN injections did not affect the expression of VGlut1 (Fig. 2a, b).

### Effects of PTN on Spontaneous Postsynaptic Currents

**Fig. 3:**
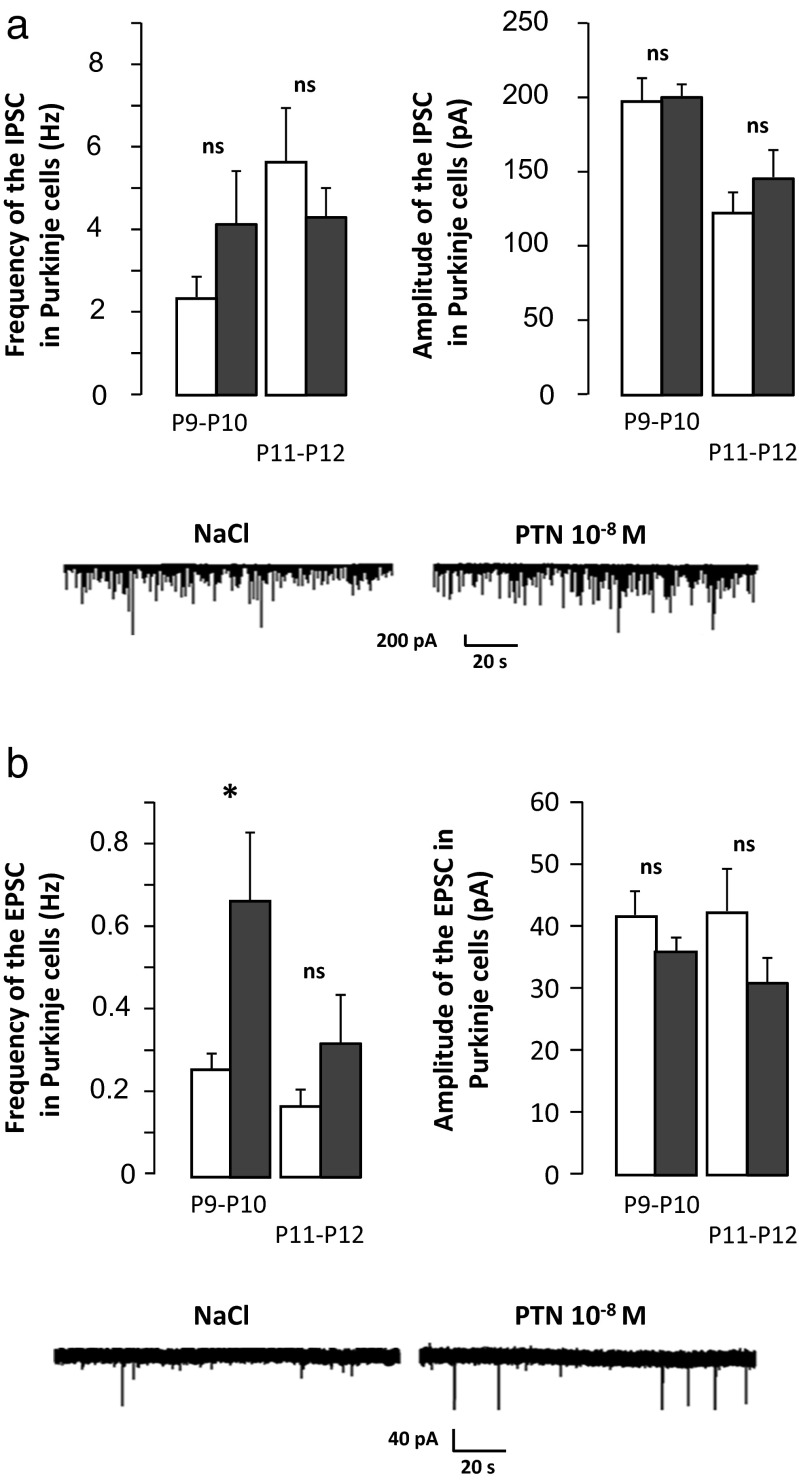
Effect of subarachnoidal injections of PTN on the spontaneous postsynaptic current on Purkinje cells during development. **a** At the *top*, measurement of the frequency (*left*) and the amplitude (*right*) of spontaneous inhibitory postsynaptic currents (IPSCs) in Purkinje cells of control (NaCl, *white bars*) and PTN-treated (PTN 10^−8^ M, *gray bars*) mice. Each value represents the mean ± SEM of the measurement of at least eight cells. At the *bottom*, representative recordings of IPSC in Purkinje cells of control (NaCl) and PTN-treated (PTN 10^−8^ M) mice at P10. **b** At the *top*, measurement of the frequency (*left*) and the amplitude (*right*) of spontaneous excitatory postsynaptic currents (EPSCs) in Purkinje cells of control (NaCl, *white bars*) and PTN-treated (PTN 10^−8^ M, *gray bars*) mice. Each value represents the mean ± SEM of the measurement of at least five cells. At the bottom, representative recordings of EPSC in Purkinje cells of control (NaCl) and PTN-treated (PTN 10^−8^ M) mice at P10. *Px* postnatal day *x*. *p < 0.05; *ns* not significant

Spontaneous inhibitory postsynaptic currents (IPSCs) and excitatory postsynaptic currents (EPSCs) respectively mediated by GABA_A_ and AMPA receptors were recorded in Purkinje cells in cerebellar slices from control and PTN-treated mice, as summarized in Fig. 3. In control mice, IPSCs displayed a frequency of 2.3 ± 0.5 Hz and an amplitude of 197 ± 15 pA (*n* = 15 cells from four animals) at P9–P10. In PTN-treated mice, no significant differences regarding either IPSC frequency or IPSC amplitude were observed (4.1 ± 1.3 Hz and 198 ± 14 pA; *n* = 9 from four animals; Fig. 3a). At P11–P12, IPSCs showed a frequency of 5.4 ± 1.5 Hz and an amplitude of 122 ± 14 pA (*n* = 8 cells from four animals). Neither the frequency (4.5 ± 0.7 Hz, *n* = 18 from four animals) nor the amplitude (140 ± 17 pA, *n* = 18 from four animals; Fig. 3a) of the IPSCs are different in PTN-treated animals. Interestingly, EPSC frequency was 2.5-fold higher in PTN-treated mice than in control animals (0.67 ± 0.17, *n* = 8 cells from two animals, vs 0.26 ± 0.04, *n* = 13 cells from three animals; p < 0.05) at P9–P10 (Fig. 3b). The same tendency was observed at P11–P12 even though the difference is not statistically significant (0.33 ± 0.10, *n* = 5 cells from three animals, vs 0.17 ± 0.04, *n* = 8 cells from four animals; Fig. 3b). It should be noted that EPSC amplitude was similar at both age in control and PTN-treated mice (P9–P10 −36 ± 3 pA, *n* = 8 cells for control, vs −42 ± 4 pA, *n* = 13 cells for PTN-treated animals; P11–P12 31 ± 4, *n* = 5 cells for control, vs −42 ± 7, *n* = 8 cells for PTN-treated animals; Fig. 3b).

### Effect of PTN Administration on the Sensory-Motor Activity in Young Animals

**Fig. 4:**
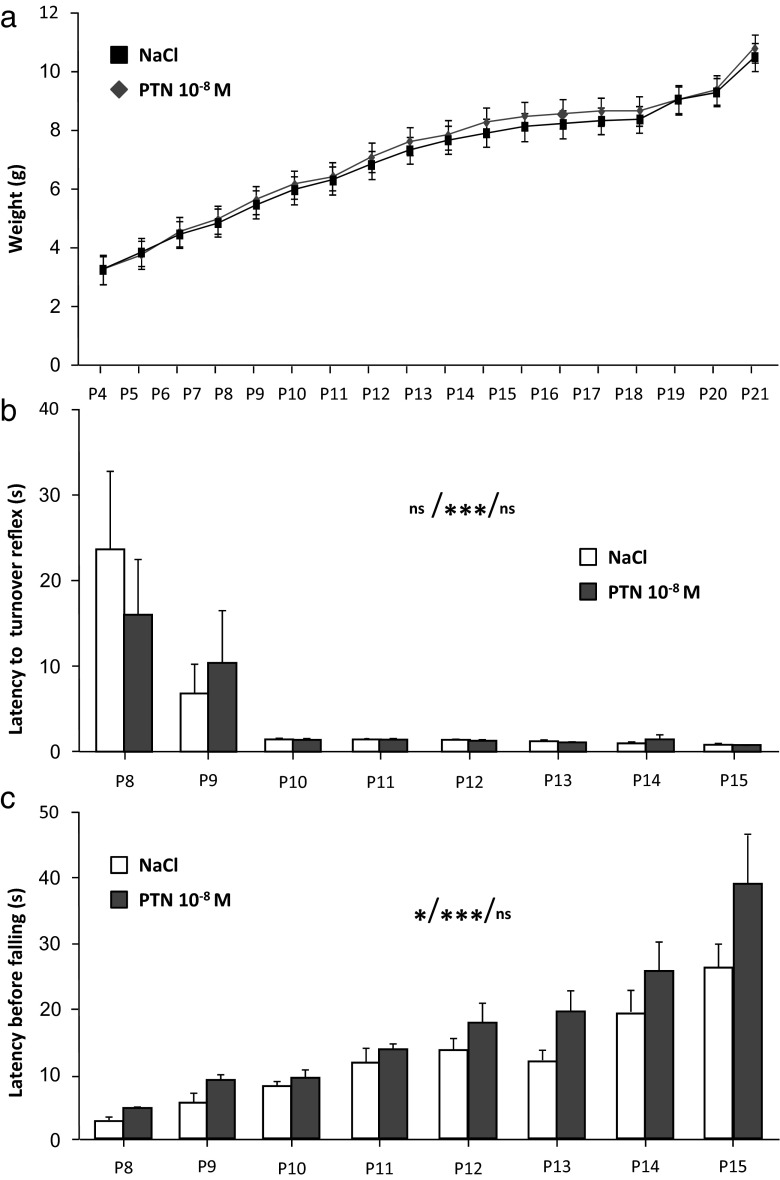
Effect of subarachnoidal injections of PTN on sensory-motor activities of mice during development. **a** Measurement of the weight gain in control (NaCl, *black squares*) and PTN-treated (PTN 10^−8^ M, *gray diamonds*) mice. **b** Measurement of the righting reflex during the two first postnatal weeks in control (NaCl, *white bars*) and PTN-treated (PTN 10^−8^ M, *gray bars*) mice. Each value represents the mean ± SEM of the performance of at least 10 animals. Statistics represent, in order, the effect of treatment, the effect of time, and the interaction between time and treatment. **c** Measurement of the grasping reflex during the two first postnatal weeks in control mice (NaCl, *white bars*) and in PTN-treated (PTN 10^−8^ M, *gray bars*) mice. Each value represents the mean ± SEM of the performance of at least 10 animals. Statistics represent, in order, the effect of treatment, the effect of time, and the interaction between time and treatment. *Px* postnatal day *x*. *p < 0.05; ***p < 0.001; *ns* not significant

The administration of PTN had no significant effect on body weight whatever their age (Fig. 4a). Between P8 and P15, the hanging test and the turnover reflex performances increased with age whatever the treatment (age *X* treatment interaction, *F*(7,133) = 0.88 and 0.411, respectively, *p* > 0.05; Fig. 4b, c). Moreover, the hanging time of PTN-treated mice was longer than the one of control animals (drug effect, *F*(1,133) = 6.22, *p* = 0.02; NaCl, *n* = 10 and PTN, *n* = 11; Fig. 4c).

At P21, our data did not reveal any statistically significant alteration of motor abilities and muscular strength in PTN-treated mice whatever the test performed (data not shown).

### Long-Term Effects of Early Postnatal Administration of PTN in Adult Mice

In all behavioral experiments, adult animals revealed neither sex nor litter differences.

#### Effect of PTN on Emotional-Related Behaviors

**Fig. 5:**
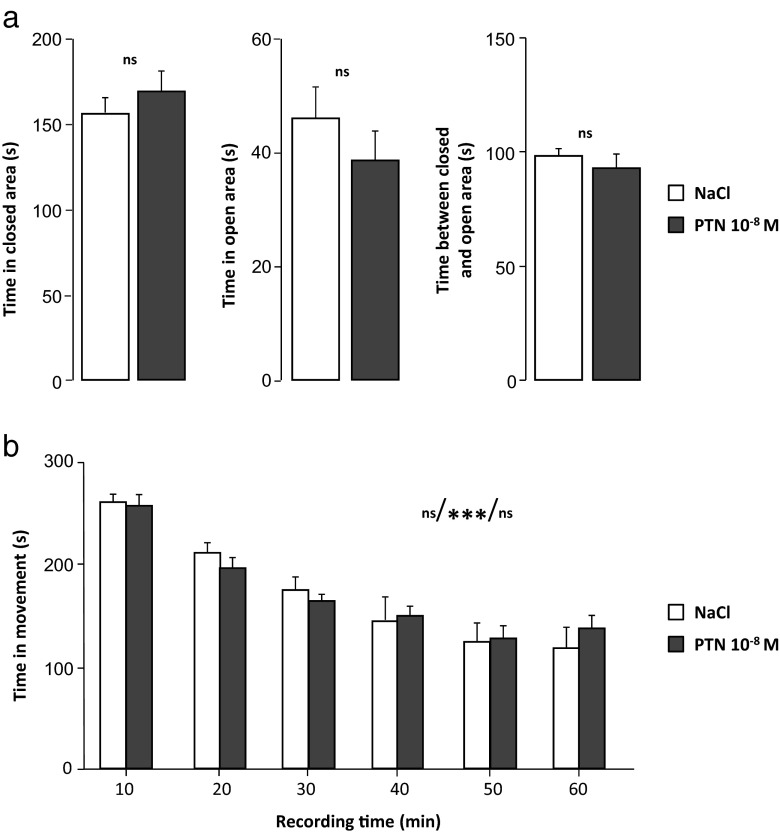
Effect of early postnatal subarachnoidal injections of PTN on the anxiety behavior and the spontaneous locomotor activity of mice at adulthood. **a** Measurement of the time spent in the closed area (*left*), in the opened area (*center*), and between the two areas (*right*) in control mice (NaCl, *white bars*) and in PTN-treated (PTN 10^−8^ M, *gray bars*) mice during the 0-maze test (5 min). Each value represents the mean ± SEM of the performance of at least seven animals. **b** Measurement of the walking time every 10 min in control mice (NaCl, *white bars*) and in PTN-treated (PTN 10^−8^ M, *gray bars*) mice during the actimetry test (60 min). Each value represents the mean ± SEM of the performance of at least seven animals. Statistics represent, in order, the effect of treatment, the effect of time, and the interaction between time and treatment. *Px* postnatal day *x*. ***p < 0.001; *ns* not significant

The results obtained during the elevated 0-maze test showed that the time spent in different areas of the apparatus was similar between the controls and PTN-treated mice (NaCl, *n* = 7 and PTN, *n* = 10; Fig. 5a).

#### Effect of PTN on Spontaneous Locomotor Activity

Neither the walking time nor the distance covered was influenced by the treatment during the whole 1-h session (data not shown). Moreover, spontaneous activity significantly decreased with time (repeated measure effect, *F*(5,75) = 45,91, *p* < 0.0001) whatever the group (interaction drug *X* repeated measures, *F*(5,75) = 0.66, *p* > 0.05; NaCl, *n* = 7 and PTN, *n* = 10; Fig. 5b).

#### Effect of PTN on Motor Coordination

**Fig. 6:**
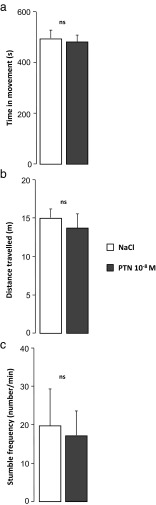
Effect of early postnatal subarachnoidal injections of PTN on the motor coordination of mice at adulthood. **a** Measurement of the walking time, **b** the covered distance, and **c** the stumble frequency in control (NaCl, *white bars*) and PTN-treated (PTN 10^−8^ M, *gray bars*) mice during the parallel rod floor test (15 min). Each value represents the mean ± SEM of the performance of at least seven animals. *ns* not significant

PTN-treated mice had a similar walking time and covered the same distance as control mice in the parallel rod floor test (NaCl, *n* = 7 and PTN, *n* = 10; Fig. 6a, b). Moreover, the two groups of animals had a similar stumble frequency (Fig. 6c).

#### Effect of PTN on Equilibrium

**Fig. 7:**
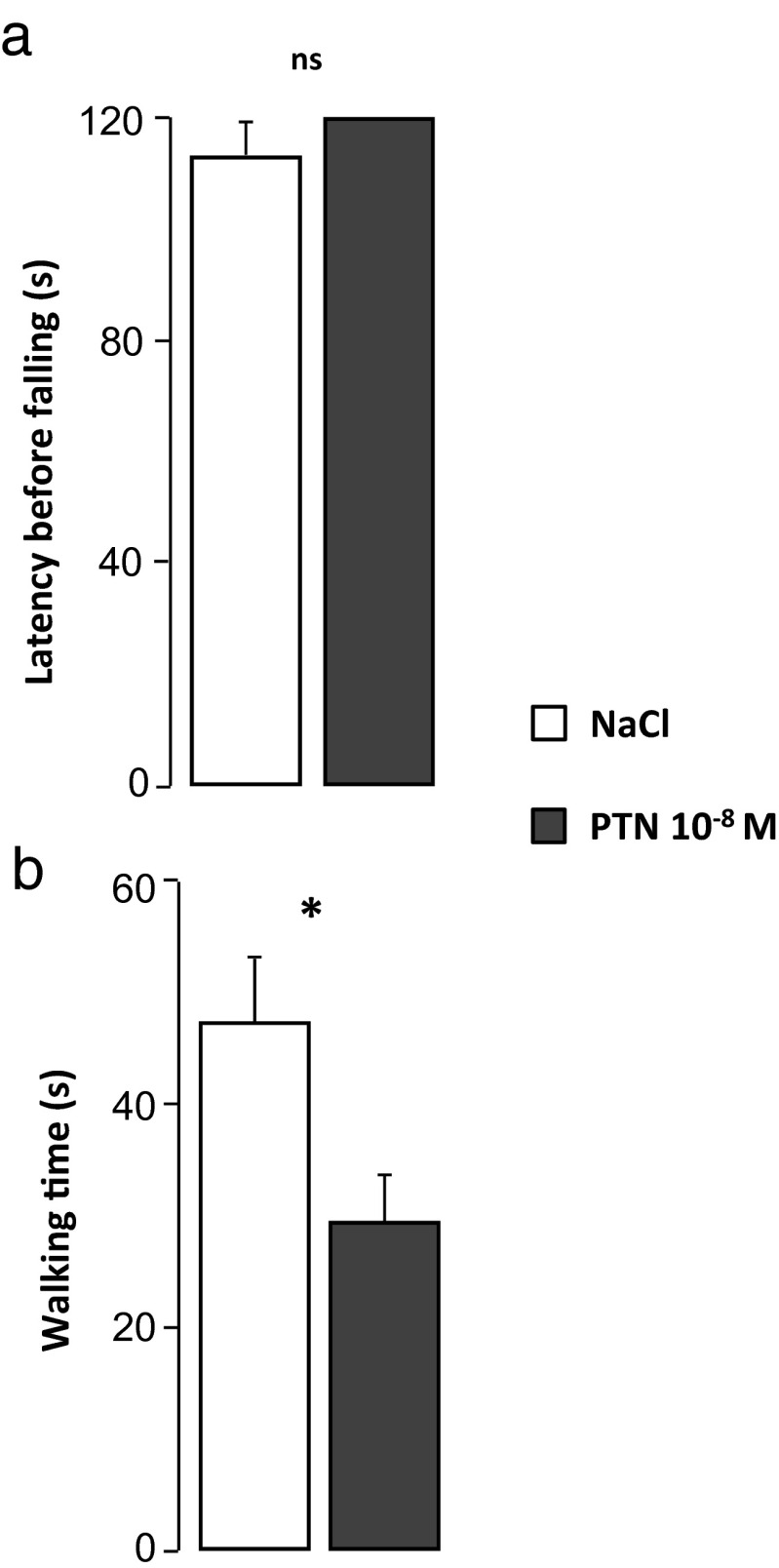
Effect of early postnatal subarachnoidal injections of PTN on equilibrium of mice at adulthood. **a** Measurement of the latency before falling in control mice (NaCl, *white bars*) and in PTN-treated (PTN 10^−8^ M, *gray bars*) mice during the unstable platform test. **b** Measurement of the walking time on the mast in control mice (NaCl, *white bars*) and in PTN-treated (PTN 10^−8^ M, *gray bars*) mice during the stationary elevated beam test (120 s). Each value represents the mean ± SEM of the performance of at least seven animals. *p < 0.05; *ns* not significant

On the unsteady platform, latencies before falling were similar in the two groups of mice, and PTN-treated mice were able to maintain balance during the complete duration of the test (NaCl, *n* = 11 and PTN, *n* = 10; Fig. 7a). Similarly, on the stationary elevated beam test, treated and control mice reached the cutoff period (data not shown). However, the time spent walking on the mast was significantly lower in PTN-treated animals compared to the control mice (*U* = 65, *p* = 0.0486; NaCl, *n* = 11 and PTN, *n* = 10; Fig. 7b), since the walking speed was similar in both groups of animals (data not shown).

#### Effect of PTN on Dynamic Motor Abilities

**Fig. 8:**
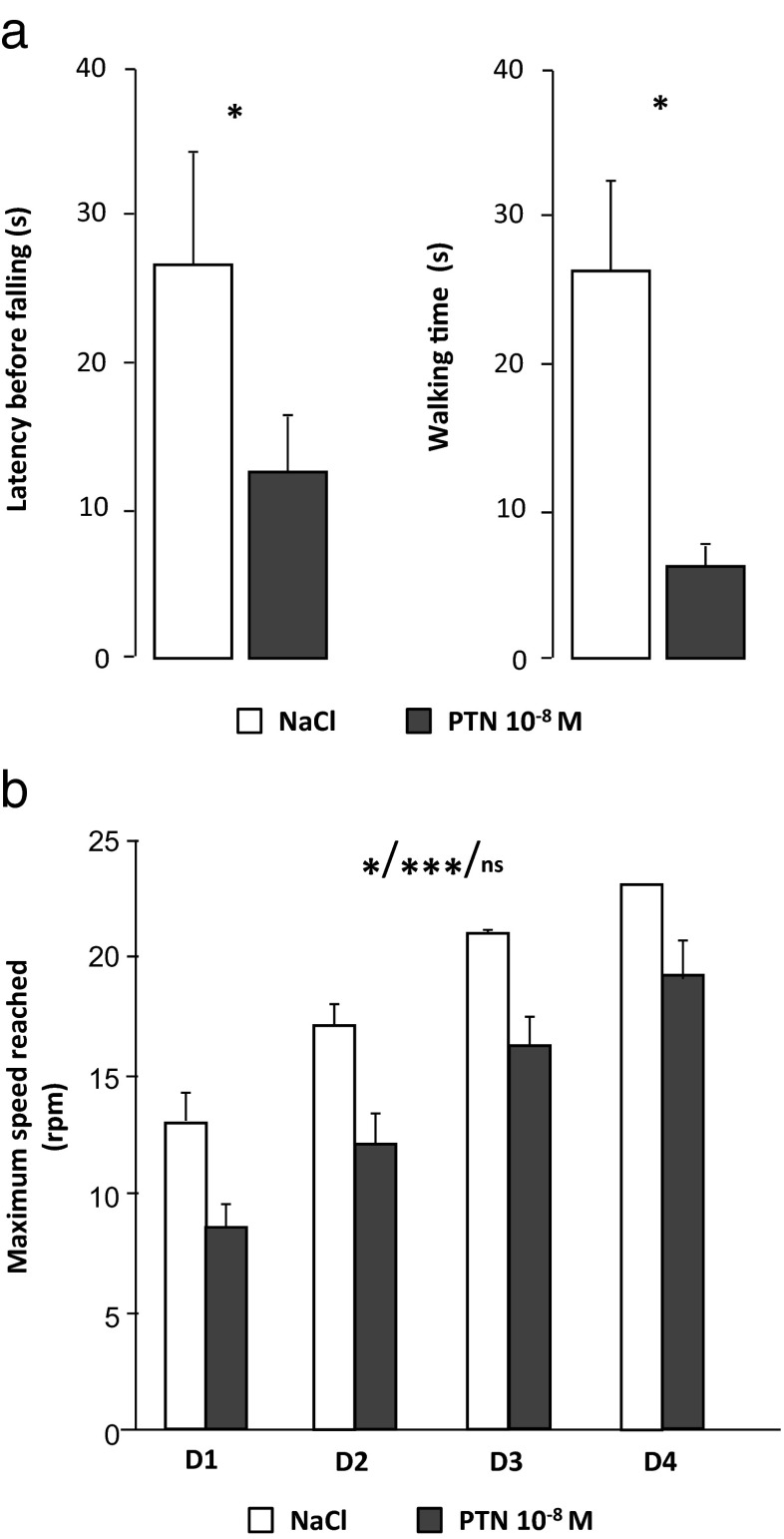
Effect of early postnatal subarachnoidal injections of PTN on the dynamic locomotor activity of mice at adulthood. **a** Measurement of the latency before falling (*left*) and the walking time (*right*) in control (NaCl, *white bars*) and PTN-treated (PTN 10^−8^ M, *gray bars*) mice during the constant speed rotarod test. Each value represents the mean ± SEM of the performance of at least seven animals. **b** Measurement of the maximal speed reached before falling in control (NaCl, *white bars*) and in PTN-treated (PTN 10^−8^ M, *gray bars*) mice during the constant accelerating rotarod test. Each value represents the mean ± SEM of the performance of at least seven animals. Statistics represent, in order, the effect of treatment, the effect of time, and the interaction between time and treatment. *Dx* day *x* of training, *rpm* rotation per minute. *p < 0.05; ***p < 0.001; *ns* not significant

The latency before falling as well as the walking time on the rotarod were significantly reduced in PTN-treated mice compared to control (*U* = 69, *p* = 0.029, and *U* = 66, *p* = 0.01, respectively; NaCl, *n* = 11 and PTN, n = 10; Fig. 8a). Furthermore, in the second device, PTN mice exhibited lower performances compared to control animals (drug effect, *F*(1,45) = 8.26, *p* = 0.0116). However, training was efficient (repeated measure effect, *F*(3,45) = 39.61, *p* < 0.0001) and similar in both groups (interaction repeated measures *X* drug, *F*(3,45) = 0.1, *p* > 0.05; NaCl, *n* = 7 and PTN, *n* = 10; Fig. 8b).

#### Effect of PTN on Gait

**Fig. 9:**
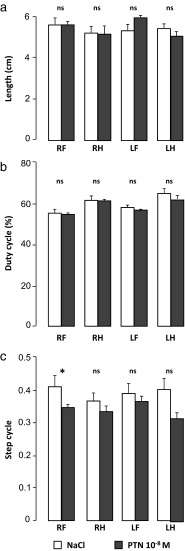
Effect of early postnatal subarachnoidal injections of PTN on the gait of mice at adulthood. **a** Measurement of the step length for each paw, **b** of the duty cycle, and **c** of the step cycle for each paw in control (NaCl, *white bars*) and in PTN-treated (PTN 10^−8^ M, *gray bars*) mice during the CatWalk test. Each value represents the mean ± SEM of the performance of at least seven animals. *RH* right hind paw, *LH* left hind paw, *RF* right front paw, *LH* left hind paw. *p < 0.05; *ns* not significant

The animals were also submitted to the CatWalk test to investigate more acutely their walking ability. As shown on Fig. 9, the distance between successive placements of each paw as well as the percentage of stance phase during the gait cycle were similar in both groups (Fig. 9a, b). However, the step cycle, which corresponds to the time between two consecutive initial contacts of the same paw, was slightly reduced, with a significant decrease for the right front paw in PTN-treated mice compared to controls (*U* = 13, *p* = 0.033; NaCl, *n* = 7 and PTN, *n* = 10; Fig. 9c).

## Discussion

The development of the cerebellum requires an appropriate microenvironment composed of various extracellular factors including molecules of the extracellular matrix. Among these, PTN, which binds the proteoglycans SDC-3 and PTP-ζ, is known to participate to the histogenesis of the cerebellar cortex. Indeed, it has been demonstrated that the in vivo administration of PTN during the first postnatal week affects the radial migration of GCP and exacerbates their apoptosis into the IGL [16]. Moreover, the perturbation of the pleiotrophinergic system during cerebellum development disrupts the morphogenesis of the dendritic tree of Purkinje cells [16, 20]. Based on these data, we investigated the consequence of PTN-induced Purkinje cell atrophy on the setting up of the cerebellar afferents and the sensory-motor activity of mice. First of all, recording and fitting of the capacitive currents of Purkinje cells revealed that PTN administration induces a significant decrease of the dendritic component of the current although its perikarya counterpart appeared not affected. These data indicate that PTN injection caused a decrease of the surface of the dendritic tree between P9 and P12 and confirmed that PTN is able to trigger an atrophy of the dendritic tree without influencing the surface of the soma [16, 27]. Since Purkinje cells represent the integrative center of the cerebellum and receive numerous afferents onto their dendrites, we examined the characteristics of both excitatory and inhibitory postsynaptic currents of PTN-treated Purkinje cells. Our data indicate that PTN administration does not modify the frequency and the amplitude of IPSC, suggesting either (i) that the global inhibitory inputs received by Purkinje cells from stellate and basket cells are not influenced by the morphology of the dendritic trees or (ii) that a putative defect of stellate cells connections on the dendrites of Purkinje cells could be compensated by an augmentation of basket cell synapses on the perisomatic component [28]. In parallel, EPSC recordings indicate that PTN administration provoked an increase of their frequency (but not the amplitude) on the Purkinje cell at P9–P10, which softened by P11–P12. Since Purkinje cells receive excitatory inputs from parallel fibers and climbing fibers, the PTN effect on EPSC frequency should be due to an increase of the synaptic transmission from one or both of these afferents. However, due to our cerebellum slicing protocol, the increase of EPSC frequency induced by PTN should be mainly attributable to an increase of the synaptic transmission from parallel fibers. So we investigate the effects of PTN on the distribution of the ionotropic glutamate receptor subunit GluRδ2 and the two vesicular glutamate transporters (VGluT1 and VGluT2) on the Purkinje cells. GluRδ2 is specifically expressed on the postsynaptic membrane of Purkinje cells apposed to parallel fiber synapses [29–31], whereas climbing fibers express VGlut2 in postnatal cerebellum [32]. VGlut2 is also present on parallel fibers during the first postnatal week, but it has been shown that its expression switches to VGlut1 from P10 in mice [32]. Immunohistochemical analysis revealed that GluRδ2 is present on 75 % of the membrane area of Purkinje cells in both control and PTN-treated mice, indicating that the number of parallel fiber contacts is proportional to the development of the Purkinje dendritic tree. This communication between granule cells and Purkinje cells has been already observed in the developing cerebellum and is known to participate to the regulation of the neuronal population in the different cortical layers [33, 34]. However, PTN treatment affects the distribution of GluRδ2 and induces a translocation of the parallel fiber connections from the dendrites to the soma of Purkinje cells. This shift of GluRδ2 localization could be due to the atrophy of the dendritic tree of Purkinje cells which precludes the proper connections of parallel fibers. In addition, the amount of VGlut2 located on the Purkinje cells is decreased in PTN-treated mice, indicating that the territory occupied by the climbing fibers is reduced. This alteration could be a direct effect of the atrophy of dendritic trees leading to shorter fibers. Moreover, it is known that the maturation of climbing fibers requires appropriate connections between the parallel fibers and the dendrites of Purkinje cells [35–37]. Thus, the disruption of GluRδ2 synaptic contacts in PTN-treated mice could induce a reduction of the contact area between climbing fibers and Purkinje cells. The deficit of afferents observed in PTN-treated mice could also explain the increase of EPSC frequency, since the current signals located close to the soma are less submitted to the attenuation process than the dendrites events [27] and are thus more frequently integrated. Moreover, the lack of climbing fiber stimulation could induce a compensatory mechanism in parallel fibers which burst to maintain a suitable excitatory rate. Since there is no difference between PTN-treated mice and controls in the EPSC amplitude, it is also conceivable that the connected parallel fibers fire with short and rapid pulses on the Purkinje cells to compensate the absence of numerous synaptic contacts.

As the Purkinje cells represent the integrative center of cerebellar neuronal circuit, we can easily imagine that the abovementioned structural and electrophysiological alteration due to perinatal PTN injection could induce short- and long-term deficits. Our results revealed that during the first postnatal weeks, PTN did not drastically disturb the development of the animals. However, the grasping scores demonstrated that the PTN treatment induces an increase of the time of maintenance between P8 and P15, whereas the muscular strength of the mice was not affected. Since the ontogenesis of the grasping reflex implicates a diminution of the muscular tone of the flexor muscles [38], this result suggests that PTN could disturb the maturation of the flexion-extension reflex allowing the release of the string.

At adulthood, the histological analysis of the calbindin immunolabeling showed a similar morphology of the dendritic tree of Purkinje cells between PTN-treated mice and controls, suggesting that a restoration of the dendritogenesis of these neurons occurred by a compensatory mechanism during late development. However, some locomotor impairment is observable in adult animals when mice were treated perinatally with PTN. Such behavioral data suggest that some misconnections must persist in adulthood. Indeed, these animals present a significant reduction of the walking time and retention time respectively on the elevated wooden mast and the rotarod apparatus, unrelated to anxiety behavior, spontaneous activity, or equilibrium deficit. Our data relative to the CatWalk test revealed a slight gait impairment (more precisely concerning the cyclic parameter) in PTN-treated mice. These behavioral data could directly result in the deficit of connections of the parallel fibers which occurred at P12. However, it has been demonstrated, in numerous transgenic mouse models, that motor coordination alterations are correlated with a default of the maturation of climbing fibers during cerebellar development [32, 39–43]. So we can envisage that the atrophy of the Purkinje cells induced by PTN during development could induce long-term alteration of climbing fiber innervation of Purkinje cells in mature cerebellum.

To conclude, our study demonstrated that an early disruption of the ECM composition induced by a subarachnoidal administration of PTN leads to significant decrease of the dendritic component of Purkinje cells associated with an increase of the EPSC frequency and an abnormal somatic localization of parallel fiber connections. These histological and electrophysiological alterations could induce a delay in the maturation of the flexion/extension reflex during development and alter locomotor coordination in adult mice.

## Abbreviations

ECM: Extracellular matrix
EGL: External granular layer
EPSC: Excitatory postsynaptic current
GCP: Granule cell precursors
GluRδ2: Glutamate receptor delta 2
IGL: Internal granular layer
IPSC: Inhibitory postsynaptic current
ML: Molecular layer
PL: Purkinje cell layer
PTN: Pleiotrophin
PTP-ζ: Protein tyrosine phosphatase zeta
P*x*: Postnatal day *x*
SDC-3: Syndecan-3
